# Histology and computed tomography of incidental calcifications in the human basal ganglia

**DOI:** 10.1007/s00234-021-02680-4

**Published:** 2021-03-20

**Authors:** Esther J. M. de Brouwer, Pim A. de Jong, Annemarieke De Jonghe, Marielle H. Emmelot-Vonk, Huiberdina L. Koek, Jan-Willem Dankbaar, Firdaus A. A. Mohamed Hoesein, Wim Van Hecke

**Affiliations:** 1 Department of Geriatrics, University Medical Center Utrecht, Room B05.2.25, PO Box 85500, 3508 GA Utrecht, Netherlands; 2 Department of Radiology, University Medical Center Utrecht, Utrecht, Netherlands; 3 Department of Geriatrics, Tergooi Hospital, Hilversum, Netherlands; 4 Department of Pathology, University Medical Center Utrecht, Utrecht, Netherlands

**Keywords:** CT scan, Basal ganglia, Pathology, Calcification

## Abstract

Incidental basal ganglia calcifications are a common finding on computed tomography (CT). We investigated the histological characteristics of these calcifications and their association with CT findings, using post-mortem basal ganglia tissue from 22 patients. Eight patients had basal ganglia calcifications on histology, and six patients had calcifications on CT, varying from mild to severe. Four patients had calcifications identified by both histology and CT, and two patients had calcifications detected by CT but not by histology, possibly because of insufficient tissue available. Calcifications were found mainly in the tunica media of arterioles located in the globus pallidus, which suggests that incidental CT calcifications are vascular in nature. However, tunica media calcifications, and thereby incidental basal ganglia calcifications, are probably not related to atherosclerosis.

## Introduction

The first radiological description of basal ganglia calcifications dates from 1924 [1]. Nowadays, calcifications in the basal ganglia are most often detected incidentally during computed tomography (CT) scanning of the brain and have a prevalence of 0.32–38% [2–4]. These calcifications are usually considered innocent, although they may be associated with diabetes and psychotic symptoms [4, 5]. Patients with Fahr disease, who have severe basal ganglia calcification, suffer from movement disorders, cognitive disorders, and psychiatric symptoms [6]. This suggests that basal ganglia calcifications may not be so harmless. In case reports of patients with Fahr disease, the calcifications occurred in capillaries and the tunica media of arterioles and small- and medium-caliber arteries, with large arteries and veins sometimes showing complete calcification of the vessel wall [7, 8]. To our knowledge, there is one neuropathological study describing the histological nature of incidental basal ganglia calcifications in patients who did not have Fahr disease but who had a neurodegenerative disease, such as Alzheimer’s disease, frontotemporal dementia, progressive supranuclear palsy, or Parkinson’s disease [9]. The study described three patterns of calcification: deposits within the tunica media, deposits in the parenchyma, and deposits along capillaries. Calcifications in the internal elastic lamina and tunica media are usually non-atherosclerotic in origin, in contrast to calcifications of the tunica intima, and are associated with diabetes mellitus and chronic kidney disease [10].

We investigated the histological nature of incidental basal ganglia calcifications and whether histological findings are associated with CT findings.

## Methods

Local ethical committee approval was obtained for research on retained tissues after written informed consent was given by the patients during life or their next of kin after death (Medical Ethics Committee of the **** 11-531/C). Between 1-1-2013 and 31-12-2018, we identified 22 adult patients for whom there were unenhanced CT scans of the brain (at maximum 1 year before autopsy) and brain autopsy findings available. Histological findings were compared to a consensus CT calcification score.

### CT scans

**Fig. 1:**
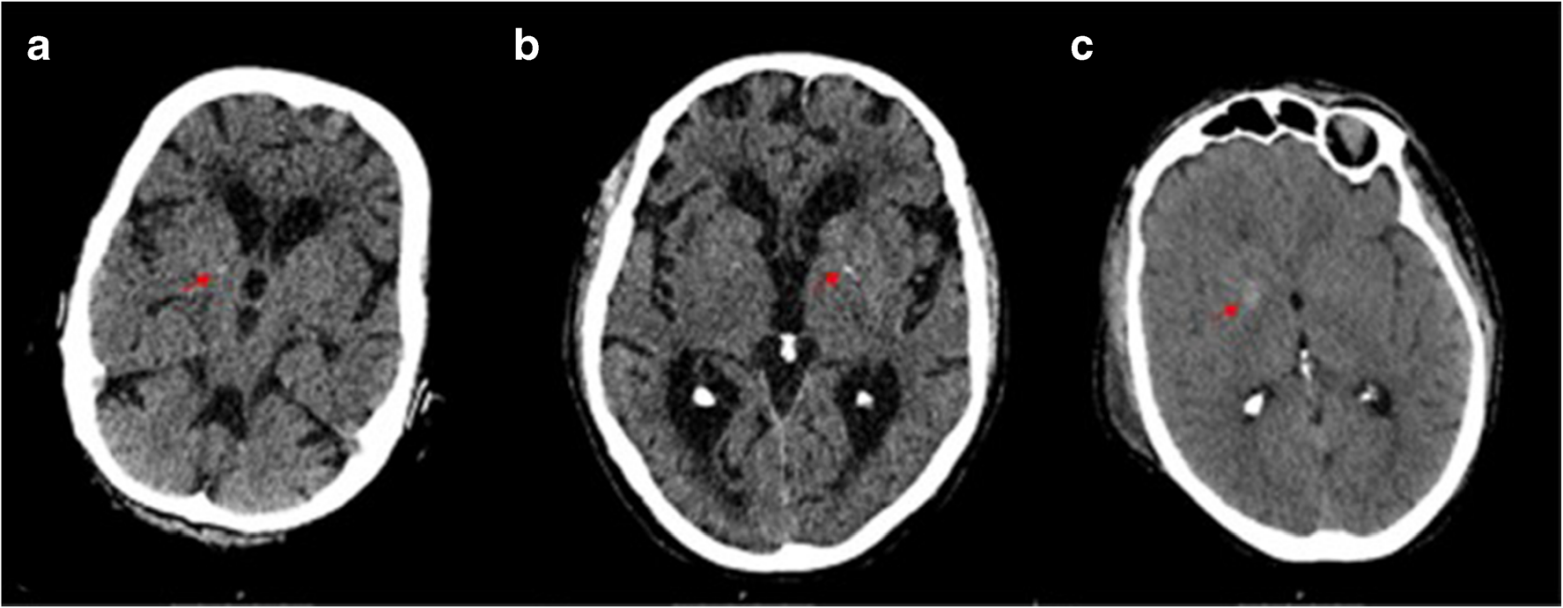
**a** CT scans with mild calcification in the right basal ganglia. **b** Moderate calcification in the left basal ganglia. **c** Severe calcification in the right basal ganglia

The 22 unenhanced CT scans of the brain were anonymized. They were acquired on Philips Brilliance 64-slice to 256-slice CT scanners (Philips Healthcare, Best, The Netherlands) and reconstructed in thin slices (max 1 millimeter). Basal ganglia calcifications were scored as absent, mild (one dot), moderate (multiple dots or a single artery), or severe (confluent) (Fig. 1) [4]. Three experienced radiologists (***, ***, ***) blinded to the histological report scored all the scans together in a consensus meeting, using the Philips IntelliSpace Portal 7.0 (Philips Healthcare, Best, The Netherlands) in the brain window setting (center 40 Hounsfield units, width 80 Hounsfield units) and axial, coronal, and sagittal views.

### Histology

**Fig. 2:**
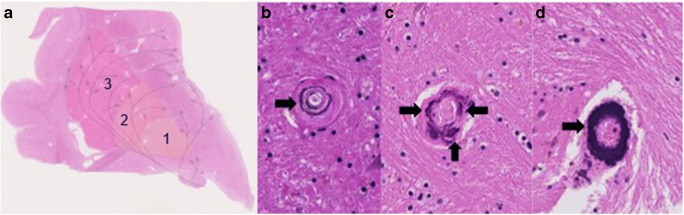
**a** Histological sections of the basal ganglia with hematoxylin staining, 1 globus pallidus interna, 2 globus pallidus externa, and 3 putamen. **b** Mild calcification. **c** Moderate calcification. **d** Severe calcification

The basal ganglia were sampled routinely and usually divided into two formalin-fixed, paraffin-embedded blocks covering the entire central grey matter up to the insular cortex. Sections were stained with hematoxylin and eosin (Fig. 2a). An experienced neuropathologist (***), who did not know the CT findings, scored the calcifications with a newly developed method as follows: mild calcification was defined as granular to fine lamellar, more often diffuse and fuchsia to purple staining; moderate calcification was defined as linear, incomplete (semi/noncircular), and deep purple staining; and severe calcification was defined as linear, circular, and deep purple to dark blue staining (Fig. 2b–d). Besides severity, the extent of calcification was assessed as being discrete/limited (sporadic), moderate (the minority), and extensive (the majority). The anatomical distribution of calcifications, i.e., globus pallidus interna, globus pallidus externa, and putamen, was assessed.

## Results

The 22 subjects (11 males) were 22–92 years old (median 69 years). On CT, six patients had calcifications, with calcification being severe in one patient, moderate in one patient, and mild in four patients (Table 1). On histology, eight patients had basal ganglia calcifications located in the tunica media of arterioles (Table 1). The severity of the calcifications was mild in three patients, moderate in three patients, and severe in two patients. The extent of the calcifications was assessed as discrete/limited in two patients, moderate in three patients, and extensive in three patients. The calcifications were localized in the internal globus pallidus in all patients and additionally in the external globus pallidus in six patients. Calcification of the putamen was not seen.

**Table 1:** Incidental basal ganglia calcifications detected by computed tomography and histology

	CT scan	Histology							
Patient number	Severity	Severity	Extent	Globus pallidus interna	Globus pallidus externa	Putamen	Age	Gender	Cause of death
1	+++	++	+++	+	+	-	56	M	Traumatic neurological injury
2	+	+++	+++	+	+	-	72	M	Pontine infarction with cerebral herniation
3	-	-	-	-	-	-	22	F	Cerebral metastatic disease
4	-	-	-	-	-	-	68	F	Pseudomembranous colitis
5	++	++	++	+	+	-	82	M	Cerebral hemorrhage with cerebral herniation
6	-	-	-	-	-	-	92	F	Subarachnoid hemorrhage
7	+	-	-	-	-	-	62	F	Ruptured aneurysm medial cerebral artery
8	+	+++	+++	+	+	-	70	F	Poor cardiopulmonary situation with bilateral pneumonia
9	+	-	-	-	-	-	71	M	Cerebral venous sinus thrombosis
10	-	+	+	+	-	-	61	F	New-onset refractory status epilepticus
11	-	-	-	-	-	-	67	M	Unknown
12	-	+	++	+	+	-	69	M	Iatrogenic rupture external iliac artery
13	-	-	-	-	-	-	56	M	Glioblastoma multiforma
14	-	++	++	+	+	-	87	F	Hypovolemic shock
15	-	-	-	-	-	-	80	F	Glioblastoma multiforma
16	-	-	-	-	-	-	53	F	Cerebral venous sinus thrombosis
17	-	-	-	-	-	-	67	M	Pulmonary sepsis
18	-	-	-	-	-	-	63	F	Thromboembolism
19	-	+	+	+	-	-	70	F	Cerebellar ischemia
20	-	-	-	-	-	-	70	M	Brainstem ischemia
21	-	-	-	-	-	-	30	M	Liver failure
22	-	-	-	-	-	-	69	M	Liver failure

The severity of calcifications of the basal ganglia detected by CT and histology was scored as absent (-), mild (+), moderate (++), or severe (+++). The histological extent of basal ganglia calcifications was scored as absent (-), in discrete/limited (+), moderate (++), and extensive (+++)

Globus pallidus interna, globus pallidus externa, and putamen: no calcification (-), presence of calcification (+). M, male; F, female

Comparison of the histological and CT findings showed that histologically proven calcifications were also seen on CT in four patients, whereas mild calcifications detected on CT were not detected histologically in two patients.

On closer inspection, the deposits in the vessel wall seemed to arise along the internal elastic lamina as granular to linear deposits in an early stage, merging with more peripheral calcifications in or along the media in a later stage, ultimately forming a single (semi-) circular deposit (Fig. 2d). This distribution pattern started in the ventral striatopallidum and fanned out posterolaterally into the external half of the globus pallidus, seemingly following the vascular tree downstream (Fig. 2a).

## Discussion

We investigated the histological nature of incidental basal ganglia calcifications and their association with CT findings. On histology, in an early stage, calcification was detected as granular to linear deposits in the internal elastic lamina, merging with more peripheral calcifications in or along the media in a later stage, ultimately forming a single (semi-) circular deposit. This is consistent with the type 1 calcifications described by Fujita [9] in patients with neurodegenerative disease and also consistent with findings in patients with Fahr disease [7, 8].

This study adds new information about calcifications in patients without Fahr disease, in group patients who underwent brain autopsy after their death.

CT scanning is the most common method to detect calcifications in the basal ganglia. However, comparison of CT and histological findings is problematic, because CT may not be sensitive enough to detect small calcifications. This is probably why CT scanning did not detect histologically proven calcifications in four patients. A limitation of histology is that sampling may miss areas with calcifications that are seen on CT, which may explain why two patients had calcifications seen on CT but not confirmed by histology. Nevertheless, in four patients, the basal ganglia calcifications seen on CT correlated with histological findings, namely, calcifications in small- and medium-sized vessels, probably following the vascular tree downstream. As the calcifications were located in the internal elastic lamina and the tunica media, we conclude that incidental CT calcifications are vascular in nature, but probably not atherosclerotic. The relevance of incidental basal ganglia calcifications needs to be investigated and correlated with motor, cognitive, and psychiatric symptoms, such as those seen in Fahr disease.
